# Wnt7a Promotes the Occurrence and Development of Colorectal Adenocarcinoma

**DOI:** 10.3389/fonc.2021.522899

**Published:** 2021-03-09

**Authors:** Congcong Li, Xiaowei Dou, Jiahuan Sun, Min Xie, Hongli Li, Peilin Cui

**Affiliations:** ^1^ Department of Gastroenterology, Beijing Tiantan Hospital, Capital Medical University, Beijing, China; ^2^ Department of Oncology, Pudong Hospital Affiliated to Fudan University, Shanghai, China; ^3^ Clinical Medical Research Center, Guizhou Medical University, Guiyang, China; ^4^ Gastrology Department, Beijing Tiantan Hospital, Capital Medical University, Beijing, China

**Keywords:** colorectal cancer, Wnt7a, occurrence, development, proliferation

## Abstract

**Objective:**

The expression of Wnt7a in colorectal cancer tissues and cell lines was analyzed, and the effect of Wnt7a on the proliferation of colorectal cancer cells was studied, so as to confirm the relationship between Wnt7a and the occurrence and development of colorectal cancer.

**Methods:**

(1) Immunohistochemical method was used to compare the expression of Wnt7a in different tissues and its relationship with the clinicopathology of colorectal adenocarcinoma. (2) The expression levels of Wnt7a in colorectal cancer cell lines HT-29 and HCT 116 were detected by qRT-PCR. (3) The down-regulated Wnt7A expression vector was constructed, and the down-regulated Wnt7A expression cell line was established. The regeneration ability of cancer cells was detected by stem cell ball formation assay, and the influence of plate cloning assay on the proliferation ability of colorectal cancer cells was detected.

**Results:**

(1) The positive rates of Wnt7a in normal colorectal mucosa, colorectal adenoma and colorectal adenocarcinoma tissues gradually increased,Wnt7a are closely related to the degree of colorectal adenocarcinoma differentiation, lymph node metastasis and Duke stage. (2) The expression level of Wnt7a in colorectal cancer cells was higher than that in normal colorectal epithelial cells. (3) The down-regulation of Wnt7A reduced the proliferation ability of colorectal cancer cells.

**Conclusions:**

Wnt7a promotes the occurrence and development of colorectal adenocarcinoma.

## Introduction

Colorectal cancer is one of the most common malignant tumors in the world. Its global comprehensive incidence rate ranks third among men in malignant tumors and second among women (1). Colorectal cancer is the second leading cause of cancer death in the word (2, 3). In China, the number of new cases of colorectal cancer and the number of cases of death has also increased year by year. From 2008 to 2013, the incidence of colorectal cancer in China has risen from 14.6/100,000 to 17.2/100,000. The mortality rate has risen from 6.18/100,000 to 7.76/100,000 (4). In China, more than 50% of patients with colorectal cancer miss the ideal time for treatment, and the 5-year survival rate is less than 40% (5). Therefore, early identification and diagnosis of colorectal cancer as well as early intervention can improve the survival rate of colorectal cancer. Several new markers have been identified as either positive or negative indicators of the disease, which indicates that complementary biomarkers may contribute to risk assessment and aid in the personalized treatment of CRC patients (6–8). Histological and serological samples are also easily collected from patients. Thus, the discovery of more prognostic biomarkers for CRC can help predict patient outcomes and provide a novel therapeutic target.

Wnt7a is closely related to a variety of tumors. (1) Wnt7a exerts a tumor suppressor effect in various cancers. Ochoa-Hernandez AB (9) demonstrated its role in leukemia, showing through the construction of overexpressing virus that Wnt7a is expressed in leukemia cell lines and Wnt7a expression in normal peripheral blood T lymphocytes is significantly higher than in leukemia cells. Calvo R (10) and Ohira T et al. (11) found that Wnt7a may play a tumor suppressor role in lung cancer (especially non-small cell lung cancer), finding that Wnt7a is down-regulated in non-small cell lung cancer. Ohira T et al. also found that Wnt7a plays an important tumor suppression role in lung cancer, which may be related to the absence of E-cadherin. (2) Wnt7a also plays a carcinogenic role. Yoshioka S et al. (12) injected Wnt7a ovarian cancer cells and Wnt7a-expressing ovarian cancer cells into nude mice, and then found that Wnt7a ovarian cancer SKOV3ip1 was knocked out. Tumor lesions and cell invasion in the cell group were relatively small. Carmon KS et al. (13) found in endometrial cancer cells by co-immunoprecipitation that Wnt7a activates the canonical Wnt/β-catenin signaling pathway by binding to Fzd5, which ultimately leads to cell proliferation. However, current studies on Wnt7a and colorectal cancer are few and preliminary.

In the present work, we analyzed the expression of Wnt7a in colorectal cancer tissues and cell lines, and studied the effect of Wnt7a on the proliferation of colorectal cancer cells, so as to confirm the relationship between Wnt7a and the occurrence and development of colorectal cancer.

## Methods

Immunohistochemical method was used to compare the expression of Wnt7a in different tissues and its relationship with the clinicopathology of colorectal adenocarcinoma.

### Patients and Tumor Specimens

Eighty specimens from the Department of Gastroenterology and General Surgery of Beijing Tiantan Hospital affiliated with Capital Medical University from June 2013 to March 2017 were collected and confirmed by pathology as colorectal adenocarcinoma. There were 49 males and 31 females; aged 24–73 years, mean age (54.88 ± 10.32) years old, 45 cases less than 65 years old, 35cases older than 65 years; 26 cases of rectal adenocarcinoma, 54 cases of colon adenocarcinoma; 51 cases with diameter ≤ 5 cm, 29 cases with diameter > 5 cm; 55 cases with infiltration into serosal layer, 25 cases without infiltration into the serosal layer; 25 cases with highly differentiated adenocarcinoma, 22 cases with moderately differentiated adenocarcinoma, 33 cases with low differentiated adenocarcinoma; 28 cases without lymph node metastasis, 52 cases with lymph node metastasis; 9 cases with distant metastasis, 71 cases with no distant metastasis; There were 32 cases with TNM stage I+II and 48 cases with TNM stage III+IV. All cases occurred with no other tumors, no tumor bleeding, intestinal perforation, intestinal obstruction, acute or chronic infection. In the control group, 20 normal tissues and 40 benign colon adenomas were selected. The appropriate paraffin tissue specimens were selected and serially sectioned 4 μm thick for immunohistochemical staining, screened by two pathologists, and the final results were confirmed.

### Immunohistochemistry Staining

Immunohistochemical analysis for Wnt7a was performed on 80 CRC specimens. The paraffin-embedded, formalin-fixed tissues were cut into 4-mm sections and dried at 70°C for 2 h. After deparaffinization and hydration, sections were rinsed in phosphate buffered saline (PBS) and then immersed in 3% hydrogen peroxide for 10 min to block endogenous peroxidase activity. Antigen retrieval was performed in boiled citrate buffer (pH 6.0) for 10 min. The slides were incubated with Wnt7a primary antibody (goat polyclonal 1:50, AF3008; R&D, Minneapolis, MN, USA) at 4°C overnight. After incubation with the secondary antibody for 30 min, sections were stained with 3,3′-diaminobenzidine (DAB) for 10 min, lightly counterstained with 10% Mayer’s hematoxylin, dehydrated, and mounted.

### Immunohistochemical Evaluation

The staining was assessed by a semi-quantitative analysis and a protein level score which is equal to the positive cell proportion score added to the cell staining score. A well-stained area was selected and observed in 10 high-power fields continuously, and more than 50 cells per field were observed. The procedure outlined in Wang Aimin, et al. (14) was then followed to calculate the proportion of stained cells. If the proportion of positive cells < 10%, the score is 0; if the proportion of positive cells is between 10% and 40% the score is 1; if the proportion of positive cells is between 40% and 70% the score is 2; if the proportion of positive cells is ≥ 70% the score is 3. The scores designate: no coloration (0 points); yellow staining (1 point); brownish yellow staining (2 points); yellowish brown staining (3 points). Final protein level scores are as follows: 0 is negative, 1–3 is +, and 4–6 is ++. Two pathologists analyzed the stained tissue sections and they were blinded to the patient’s clinical parameters.

### Western Blot

Total protein was extracted and lysed with RIPA buffer containing protease inhibitor cocktail (Roche). The proteins were separated by SDS-PAGE electrophoresis and transferred to membrane. Membranes were probed with specific primary antibodies against β-actin (Santa Cruz), Wnt7a (abcam), and detected by horseradish peroxidase-conjugated secondary antibodies.

The expression levels of Wnt7a in colorectal cancer cell lines HT-29 and HCT 116 were detected by qRT-PCR.3. The down-regulated Wnt7A expression vector was constructed, and the down-regulated Wnt7A expression cell line was established. The regeneration ability of cancer cells was detected by stem cell ball formation assay, and the influence of plate cloning assay on the proliferation ability of colorectal cancer cells was detected.

### Cell Lines

Two human colorectal cancer cell lines HT-29, HCT 116 cells were purchased from the Chinese Academy of Sciences Cell Bank (Shanghai, China). A normal colorectal epithelial cell line, HCOEPIC, was purchased from a laboratory in Carlsbad, California, USA.

### Cell Culture

Colorectal cancer cell lines HT-29 and HCT 116 were cultured in a constant temperature and humidity incubator containing 5% CO_2_ at 37°C using complete medium (containing McCoys5A medium, 10% fetal bovine serum, 100 U/ml penicillin and 100 ug/ml streptomycin). in. According to the cell growth condition, the medium was cultured once every 2–3 days, and when the cells covered most of the surface of the bottom wall of the bottle, the cells were passaged or collected.

### Vector Construction and Lentivirus Synthesis

Expression of Wnt7a was inhibited by small hairpin RNA (shRNA) technology constructing a lentiviral vector. The lentivirus vector for silencing Wnt7A were ordered from sigma. The target sequence for shRNA were: shWnt7A-b: GCGTTCACCTACGCCATCATT; shWnt7A-c: CATAGGAGAAGGCTCACAAAT. The sequence coding human Wnt7A was amplified by reverse transcriptase polymerase chain reaction (RT-PCR): (forward primer: 5’-TTGGCGCGCCGCCACCATGAACCGGAAAGCGCGGCG-3’ and reverse primer: primers: 5’-CCTTAATTAATCACTTGCACGTGTACATCT-3’) and inserted into the AscI/PacI sites of pCDH. The shRNA or pCDH were cotransfected with packaging vector psPAX2 and MD2G overnight and changed medium. 48 and 72 h after transfection, the viral was collected and infected HT29 or HCT116 cells in the presence of 8 ug/ul polybrene overnight. The cells for silencing Wnt7a were selected by puromycin.

### qRT-PCR

Total RNA was isolated using Trizol reagent (Invitrogen), and reverse-transcription wre performed using PrimeScript™ RT reagent Kit (Takara) according to manufacturer’s instructions. Real-time PCR were performed using SYBR Premix Ex Taq II (Takara). The following primers were used: Wnt7A (5’-CTGTGGCTGCGACAAAGAGAA-3’ and 5’-GCCGTGGCACTTACATTCC-3’), CyclinD1 (5’-GCTGCGAAGTGGAAACCATC-3’ and 5’-CCTCCTTCTGCACACATTTGAA-3’) and Cyclin E1 (5’- AAGGAGCGGGACACCATGA-3’ and 5’-ACGGTCACGTTTGCCTTCC-3’). The primer for internal control β-actin (5’-GAGACCTTCAACACCCCAGCC-3’ and 5’- AATGTCACGCACGATTTCCC-3’). The Real-time PCR were carried out by the CFX96 Real-time PCR Detection System (BIO-RAD). Each sample was done in triplicate and β-actin was used as a reference for normalization. The relative expression level was evaluated through Normalized Gene Expression (ddCT) by the CFX96 data analysis software.

### Sphere Formation Assay

The colorectal cancer cells (HT-29 HCT 116) were digested with typsine and filtered with a 40-um strainer. The cells were grown in serum-free DMEM/F-12 (Gibco) supplemented with 10 ng/ml human recombinant bFGF (basic fibroblast growth factor; R&D), 10 ng/ml EGF (epidermal growth factor; Gibco) and B27 (Gibco). The cells were cultured in 2 ml medium in each well of a 6-well ultralow attachment plate and medium was added every 3 days. The size of tumorsphere were evaluated after 7 days of culture. Each experiment was performed in triplicate and measured by two investigators.

### Plate Clone Formation Assay

The cells in the logarithmic growth phase were digested with 0.25% trypsin and blown into individual cells, and the cells were suspended in DMEM medium containing 10% fetal bovine serum for use. 2. The cell suspension was diluted as a gradient. Each group of cells was inoculated with 10 mL of 37°C pre-warmed culture medium at a gradient density of 50, 100, and 200 cells per dish, and gently rotated to disperse the cells evenly. It was then incubated in a cell culture incubator at 37°C 5% CO_2_ and saturated humidity for 2 to 3 weeks. 3. It is often observed that when macroscopic clones appear in the culture dish, the culture is terminated. The supernatant was discarded and carefully immersed twice in PBS. Add 4% paraformaldehyde fixed cells to 5 mL for 15 min. Then, the appropriate amount of GIMSA application staining solution was for 10 to 30 min, and slowly washed away with running water and air dried. 4. The plate was inverted and a grid of transparencies was overlaid. The clones were counted directly. Finally, the clone formation rate was calculated. Clonal formation rate = (number of clones/number of cells inoculated) × 100%.

### Statistical Analysis

SPSS 19.0 was used for statistical analysis. Measurement data that conform to the normal distribution are expressed as mean ± standard deviation (x ± s), independent sample t-test or one-way ANOVA were used for comparison between groups, and non-normal distribution is expressed by median and interquartile range; In terms of percentage, the comparison between the positive rates was performed by χ2 test; the correlation analysis of the two variables was performed by χ2 test or Spearman correlation analysis, the test level was 0.05, and the two-sided test was used. P < 0.05 was statistically significant.

## Results

(1) The expression of Wnt7a was assessed in normal colonic mucosa, colorectal adenoma and colorectal cancer tissues.

**Figure 1:**
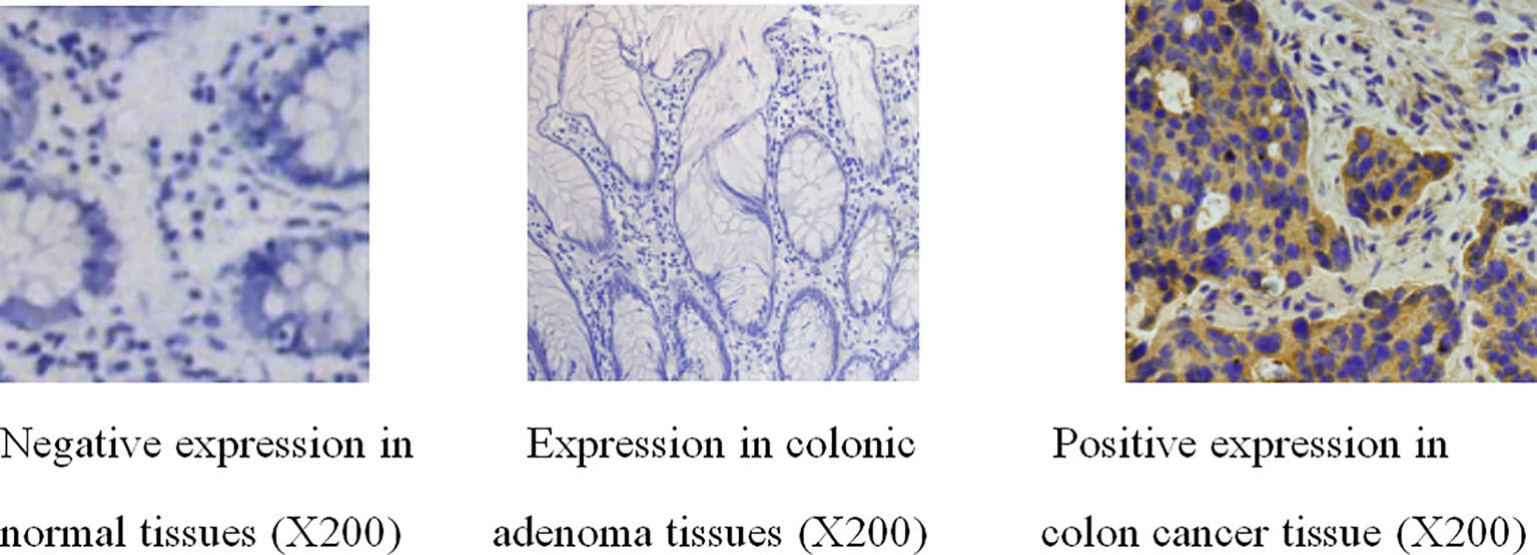
Immunohistochemical results of Wnt7a in normal tissues, colonic adenoma tissues and colonic adenocarcinoma tissues.

The positive expression rate of Wnt7a in colorectal adenocarcinoma was significantly higher than that in colorectal adenoma and normal colorectal mucosa (P<0.01) (**Table 1**) (**Figure 1**).

**Table 1:** Expression of Wnt7a in three different colon tissues.

Tissue type	N	Wnt7a protein positive expression rate		Positive rate (%)	P
		Positive	Negative		
Normal mucosal tissue	20	4	16	20	
Colorectal adenoma	40	13	27	32.5	0.31
Colorectal adenocarcinoma	80	54	26	67.5	0.00
χ^2^	21.876				
P	< 0.01*				

*indicates a P value less than 0.05.

(2) Correlation between immunohistochemical wnt7a expression and clinicopathological.

The positive expression of Wnt7a protein was not related to the gender, age, lesion location, path length, depth of invasion, and distant metastasis (P > 0.05), but was related to the TNM stage, cell differentiation, and lymph node metastasis. Clinical pathological parameters were associated (P < 0.01). The lower the degree of differentiation, the higher the stage and the colorectal cancer with lymph node metastasis, the higher the positive rate of Wnt7a (**Table 2**).

**Table 2:** Correlation between Wnt7α expression and clinicopathological information in primary CRC.

Characteristic	No. of cases	Wnt7a					
		−	+	++, +++	Positive rate	χ^2^	P
Gender							
Male	49	14	8	27	71.4%	0.890	0.346
Female	31	12	6	13	61.3%		
Age (years)							
< 65	45	15	10	20	66.7%	0.033	0.857
≥ 65	35	11	9	15	68.6%		
location							
Colon	26	10	5	11	61.5%	0.624	0.430
Rectu	54	16	9	29	70.4%		
Tumor size							
≤ 5 cm	51	18	9	24	64.7%	0.501	0.479
> 5 cm	29	8	6	15	72.4%		
Depth							
Undiluted serosa	25	10	6	930	60%	0.932	0.334
Infiltrated serosa	55	16	9	30	70.9%		
Histological grade							
Well/moderate	47	21	20	6	55.3%	7.706	0.005*
Poor	33	5	7	21	84.8%		
Lymph node metastasis							
Present	52	9	7	36	82.7%	15.631	0.00*
Absent	28	17	2	9	39.3%		
Vascular invasion							
Present	9	3	1	5	66.7%	0.003	0.955
Absent	71	23	10	38	67.6%		
TNM staging							
I+II	32	15	4	13	53.1%	5.024	0.025*
III+IV	48	11	8	29	77.1%		

*indicates a P value less than 0.05.

**Figure 2:**
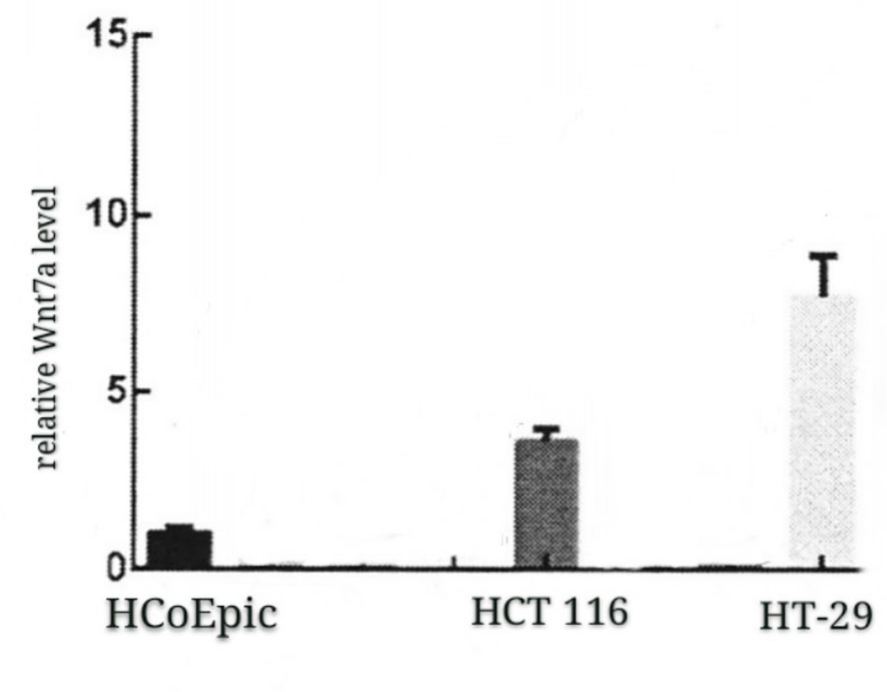
The expression level of Wnt7a in HT-29, HCT 116 and normal colorectal epithelial cell line, HCoEpic was detected by qRT-PCR.

(3) The expression level of Wnt7a in colorectal cancer cell lines HT-29 and HCT 116 was higher than that in normal colon epithelial cell lines HCoEpic (**Figure 2**).

(4) The knockout of Wnt7A gene inhibited the proliferation of colon cancer cells.

**Figure 3:**
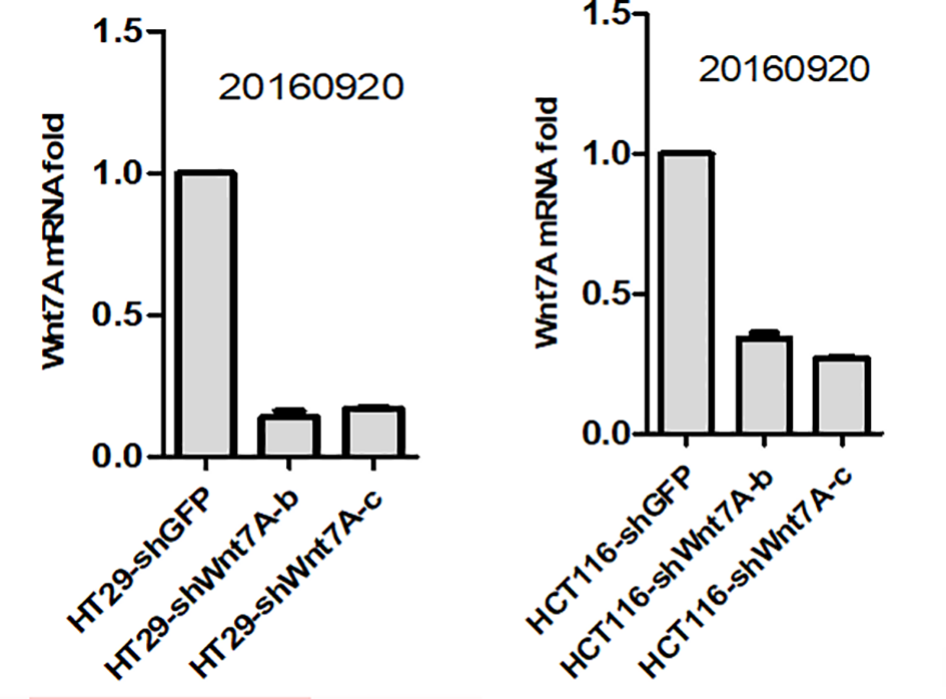
Real-time PCR confirmed that shWnt7b and shWnt7c effectively silenced Wnt7a expression at protein and mRNA level.

**Figure 4:**
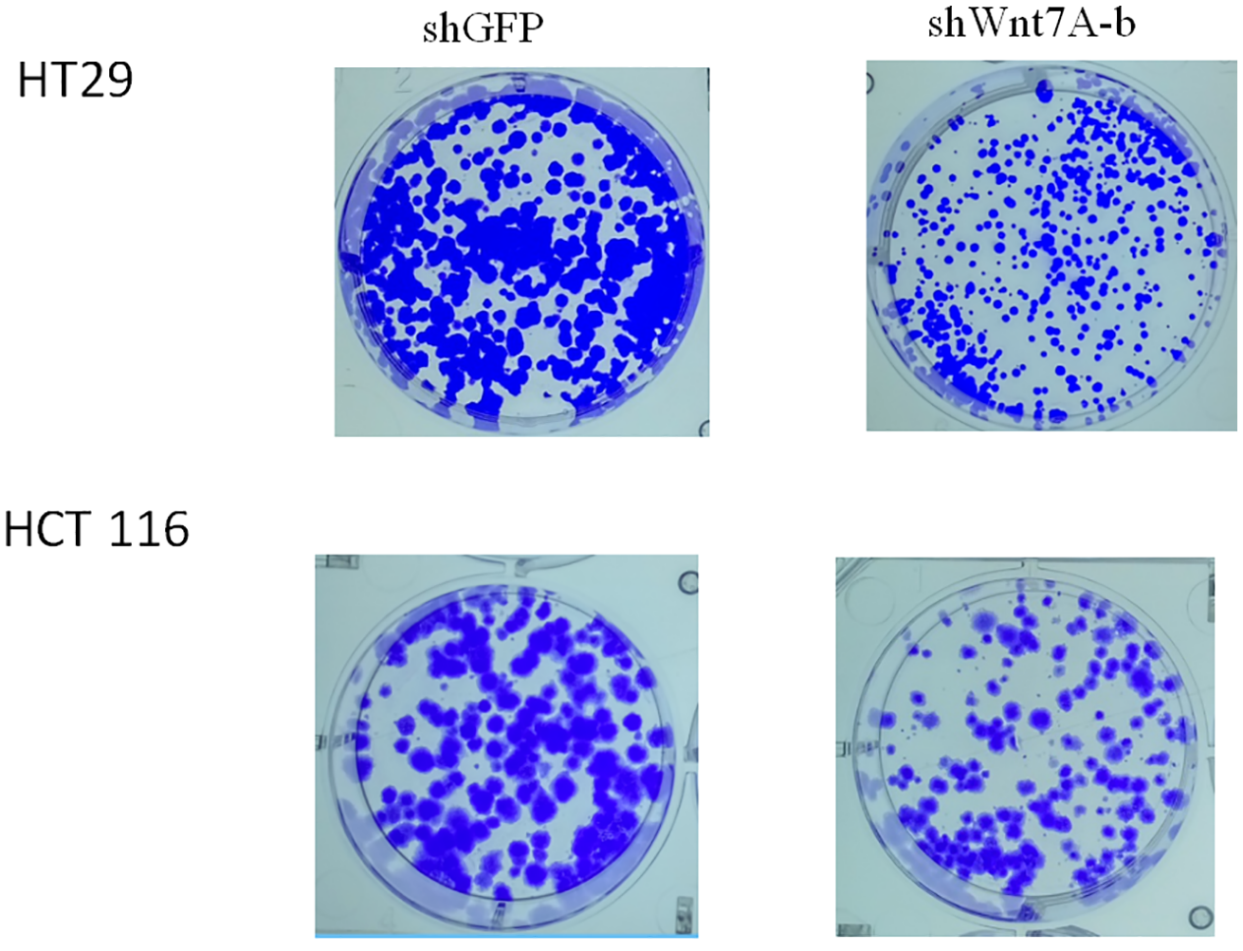
Colony formation assay.

**Figure 5:**
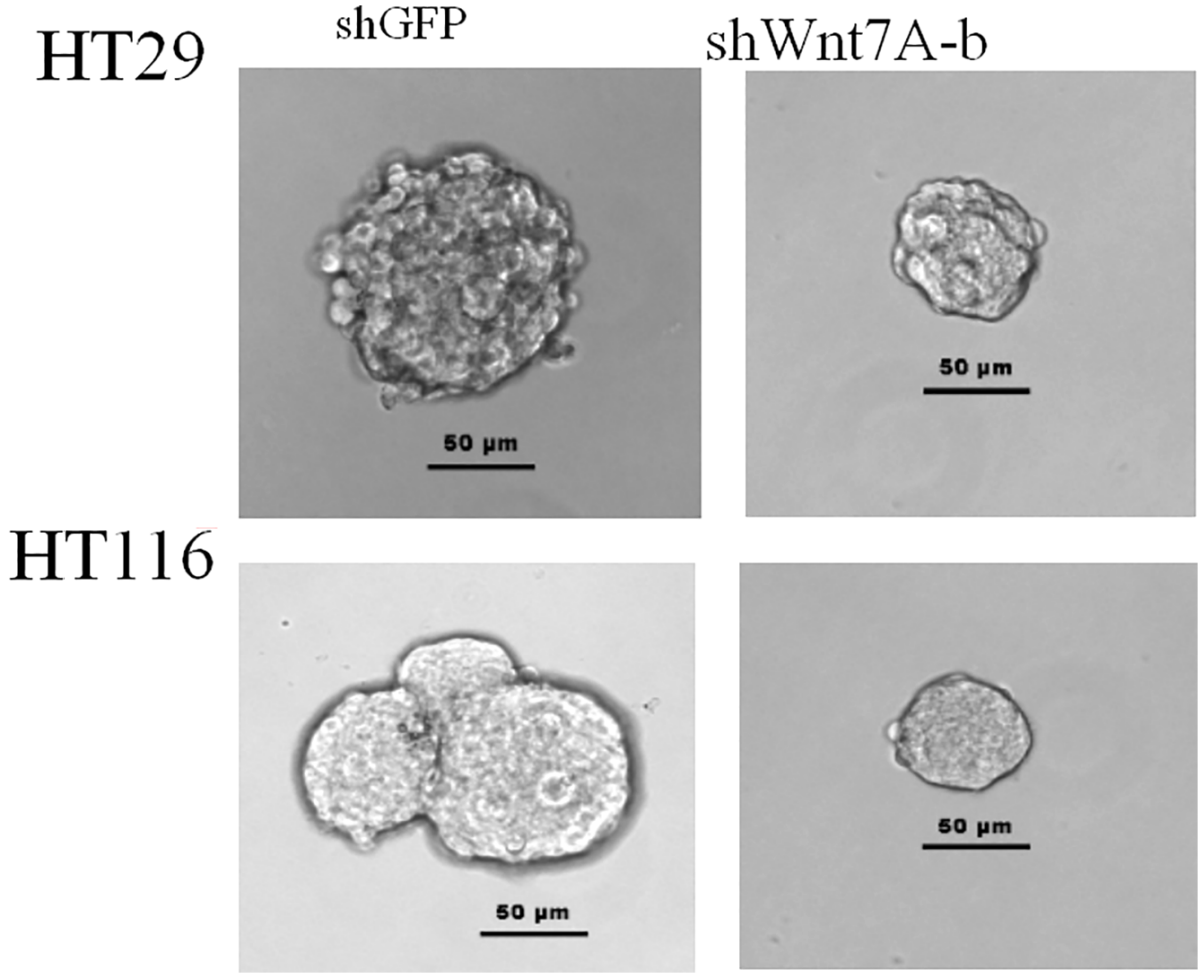
Sphere formation assay.

To investigate the effect of Wnt7a on the proliferation of colorectal cancer cells, we first silenced Wnt7a in colon cancer cell lines HT29 and HT116. Real-time PCR confirmed that SHWNT7B and SHWNT7C effectively silenced WNT7A expression at the protein and mRNA levels (**Figure 3**).After culture with pellet forming medium, Wnt7A knockout reduced the number of tumor globules in HT29 and HT116 cells. Clonal formation assay was used to detect the effect of knockdown Wnt7a on colonization ability of colon cancer cells. After knockdown of Wnt7A, the number of clone formation of HT-29 and HCT 116 of colon cancer cells was significantly reduced compared with the control group. These results suggest that silencing Wnt7a inhibits colon cancer proliferation (**Figures 4** and **5**).

## Discussion

In recent years, with the change of people’s lifestyle, the strengthening of awareness of early diagnosis and early treatment, as well as the gradual progress of colorectal cancer surgery, radiotherapy and chemotherapy technology, the 5-year survival rate of colorectal cancer is still only 66.1%, and if distant metastasis occurs, the 5-year survival rate of patients is as low as 12.5% (15). The high morbidity and mortality of colorectal cancer have a great impact on the social and family life of patients and their families. How to find the biomarkers that can diagnose colorectal cancer early and predict the prognosis of colorectal cancer has become a research hotspot around the world.

Wnt7a is an important protein in the Wnt signaling pathway. A number of studies have shown that Wnt7a plays an oncogenic or anticancer role in a variety of tumors. Currently, the studies on Wnt7a and colorectal cancer are still in the preliminary stage, so the study on its influence on the occurrence and development of colorectal cancer may provide a new idea for the diagnostic treatment of colorectal cancer.

The conclusion of this study suggests that the positive expression of Wnt7a in colon adenocarcinoma is significantly higher than that in colorectal adenoma and normal colorectal mucosa. In addition, by studying the relationship between Wnt7a and clinical pathological parameters of colorectal cancer, it suggests that Wnt7a can be used as a reference index to evaluate the malignant degree and metastasis of colorectal cancer, and provide a reference for the prognosis of colorectal cancer. However, the number of experimental studies is small, the results of research may have some deviation, and follow up studies have not been done. Therefore, the prognosis cannot be accurately evaluated and further optimization is needed. In addition, to investigate the potential role of Wnt7a in colon cancer tumorigenesis, Wnt7a was silenced in the colon cancer cell lines HT29 and HT116. Real-time PCR confirmed that shWnt7b and shWnt7c effectively silenced Wnt7a expression at mRNA levels. Thus, we surmise that Wnt7α plays a key role in the transformation process from adenoma to carcinoma by promoting cancer cell growth and metastasis. In this study, siRNAs specific to Wnt7A were transfected into 2 colon cancer cell lines. Real-time PCR was used to verify the knockdown efficiency. Then, sphere formation experiment and plate clone formation experiment were carried out *in vitro* to verify the effect of Wnt7a on the growth of colon cancer cells. After knockdown of Wnt7A, the proliferation rate of colorectal cancer cells was slowed down and the ability of clone formation was reduced, which proved that Wnt7A could promote the growth of colorectal cancer cells.

The results of this study only reveal associations, and further study is necessary to investigate the potential mechanism of Wnt7a in CRC development.

## Data Availability Statement

The data used during the current study are available from the corresponding author on reasonable request.

## Ethics Statement

This study was approved by the Medical Ethics Committee of Beijing Tiantan Hospital, Capital Medical University, Beijing, China. Written informed consent to participate in this study was obtained from all patients at the time of admission.

## Author Contributions

CL and XD contributed equally to this work. All authors contributed to the article and approved the submitted version.

## Conflict of Interest

The authors declare that the research was conducted in the absence of any commercial or financial relationships that could be construed as a potential conflict of interest.

## References

[1] Nusse R Brown A Papkoff J Scambler P Shackleford G McMahon A. A new nomenclature for int-land related genes,the wnt gene family. Cell (1991) 64:231–2. 10.1016/0092-8674(91)90633-A 1846319

[2] Hsieh JC Kodjabachian L Rebbert ML Rattner A Smallwood PM Samos CH. A new secreted protein that binds to Wnt proteins and inhibits their activities. Nature (1999) 398(6726):431–6. 10.1038/18899 10201374

[3] Dann CE Hsieh JC Ratmer A Sharma D Nathans J Leahy DJ. Insights into Wnt binding and signalling from the structures of two Frizzled cysteine-rich domains. Nature (2001) 412(6842):86–90. 10.1038/35083601 11452312

[4] Willert K Brown JD Danenberg E Duncan AW Weissman IL Reya T. Wnt proteins are lipid-modified and Can act aS stem cell growth factors. Nature (2003) 423(6938):448–52. 10.1038/nature01611 12717451

[5] RAO TP KUHL M. An updated overview on Wnt signaling pathways: a prelude for more. Circ Res (2010) 106(12):1798–806. 10.1161/CIRCRESAHA.110.219840 20576942

[6] Xu J Wan XB Huang XF Chan KC Hong MH Wang LH. Serologic antienzyme rate of Epstein-Barr virus DNase-specific Neutralizing antibody segregates TNM classification in nasopharyngeal carcinoma. J Clin Oncol (2010) 28:5202–9. 10.1200/JCO.2009.25.6552 21060035

[7] Fan XJ Wan XB Fu XH Wu PH Chen DK Wang PN. Phosphorylated p38, a negative prognostic biomarker, complements TNM staging prognostication in colorectal cancer. Tumour Biol (2014) 35:10487–95. 10.1007/s13277-014-2320-3 25056534

[8] Fang Y Jiang Y Wang X Yang X Gao Y Wang J. Somatic mutations of the HER2 in metastatic breast cancer. Tumour Biol (2014) 35:11851–4. 10.1007/s13277-014-2414-y 25326805

[9] Ochoa-Hernandez AB Ramos-Solano M Meza-Canales ID Garcia-Castro B Rosales-Reynoso MA Rosales-Avina JA. Peripheral T-lymphocytes express WNT7A and its restoration in leukemia-derived lymphoblasts inhibits cell proliferation. BMC Cancer (2012) 12:60. 10.1186/1471-2407-12-60 22313908 PMC3299642

[10] Calvo R West J Franklin W Erickson P Bemis L Li E. Altered HOX and WNT7A expression in human lung cancer. Proc Natl Acad Sci USA (2000) 97(23):12776–81. 10.1073/pnas.97.23.12776 PMC18840 11070089

[11] Ohira T Gemmill RM Ferguson K Kusy S Roche J Brambilla E. WNT7a induces E-cadherin in lung cancer cells. Proc Natl Acad Sci USA (2003) 100(18):10429–34. 10.1073/pnas.1734137100 PMC193578 12937339

[12] Yoshioka S King ML Ran S Okuda H MacLean J McAsey M. WNT7A Regulates Tumor Growth and Progression in Ovarian Cancer through the WNT/β-Catenin Pathway. Mol Cancer Res (2012) 10(3):469–82. 10.1158/1541-7786.MCR-11-0177 PMC3307825 22232518

[13] Carmon KS Loose DS. Secreted frizzled-related protein 4 regulates two Wnt7a signaling pathways and inhibits proliferation in endometrial cancer cells. Mol Cancer Res (2008) 6(6):1017–28. 10.1158/1541-7786.MCR-08-0039 18567805

[14] Wang A Jiao H Wu J Wang X Li J. Expression changes of EGFR, c-Met and Ki67 in gastric cancer and their clinical significance. J Shandong Med (2017) 57(8):75–6. 10.3969/j.issn.1002266X.2017.08.024

[15] Siegel R Desantis C Jemal A. Colorectal cancer statistics, 2014. Ca-a Cancer J Clinicians (2014) 64(2):104–17. 10.3322/caac.21220 24639052

